# Invasive Mucinous Adenocarcinoma in a Newborn With Antenatally Diagnosed Congenital Pulmonary Airway Malformation: A Case Report

**DOI:** 10.7759/cureus.66063

**Published:** 2024-08-03

**Authors:** Madhura Gopalaswamy, Ferwa Asif, Abdullah Alshammari, Mark Boyle, Silviu Buderi, Thomas Semple, Yu Zhi Zhang, Andrew G Nicholson, Simon Jordan

**Affiliations:** 1 Cardiothoracic Surgery, Royal Brompton Hospital, London, GBR; 2 Thoracic Surgery, Royal Brompton Hospital, London, GBR; 3 Surgery and Cancer, Imperial College London, London, GBR; 4 Radiology, Royal Brompton Hospital, London, GBR; 5 Histopathology, Royal Brompton Hospital, London, GBR

**Keywords:** congenital lung malformations, neonate, lung lobectomy, ecmo, cpam

## Abstract

Congenital pulmonary airway malformations (CPAMs) are rare multicystic lung lesions typically diagnosed antenatally. We present a case of a term female neonate with antenatally diagnosed CPAM who required pleuro-amniotic shunting at 22 weeks of gestation. The patient was born with a right-sided pneumothorax and severe cardiorespiratory distress, necessitating extracorporeal membrane oxygenation (ECMO). Chest CT confirmed CPAM, revealing multiple cystic lesions in the right middle lobe and a significant contralateral mediastinal shift. On the second day of life, while on ECMO, the patient underwent a right middle lobectomy and an upper lobe anterior segmentectomy via a posterolateral thoracotomy. Post-surgery cardiac CT showed narrowing of the left pulmonary artery, although a perfusion study indicated normal left lung perfusion. Histopathological examination identified CPAM type 1 with invasive mucinous adenocarcinoma (IMA; stage 1: pT1b), featuring low-to-intermediate cellularity and KRAS G12D mutations. The invasive mucinous component measured at least 15 mm but did not invade the visceral pleura. After a gradual weaning process, the patient was successfully extubated and discharged home after 70 days. To our knowledge, this is the first reported case of CPAM type 1 with IMA that underwent pleuro-amniotic shunting in the second trimester.

## Introduction

Congenital pulmonary airway malformations (CPAMs) are rare developmental cystic lung lesions characterized by architectural disorganization of the respiratory structures [1]. Large-scale population studies from the UK, Canada, and Hong Kong estimate an incidence of 4 per 10,000 live births [2-5]. Stocker described five major types of CPAMs (Appendix A), each originating from different parts of the bronchial tree. CPAM type 1, the most common, consists of bronchiole-like cysts up to 20 mm in diameter, along with smaller cysts and alveolar parenchyma [6,7].

In approximately one-third of type 1 CPAM cases, proliferation of cytologically bland mucinous cells is observed, with terms such as mucinous cell hyperplasia, mucinous metaplasia, and atypical goblet cell hyperplasia being used to describe these changes [8-10]. These mucinous proliferations can occasionally extend along the alveolar septa into adjacent alveoli, making them difficult to differentiate from invasive mucinous adenocarcinoma (IMA) or mucinous adenocarcinoma in situ (AIS), depending on factors like lesion size, invasion, and multifocality [11]. Molecular analysis of these mucinous cells has revealed various mutations, including KRAS, EGFR, and others, which are similar to those commonly found in de novo mucinous adenocarcinomas in adults [12].

We present a case of IMA in a newborn with antenatally diagnosed CPAM type 1, who had undergone in-utero pleuro-amniotic shunting.

## Case presentation

We report a case of antenatally diagnosed CPAM. During antenatal visits in the second trimester, an ultrasound revealed a large solid and cystic lesion in the right hemithorax, leading to a provisional diagnosis of large-mixed CPAM. At 22 weeks of gestation, a fetal pleuro-amniotic shunt was inserted, placing a tube between the fetal thorax and the amniotic cavity to prevent fetal hydrops. The shunt was removed shortly after birth. Obstetric consultation recommended an elective cesarean section at 39 weeks.

**Figure 1:**
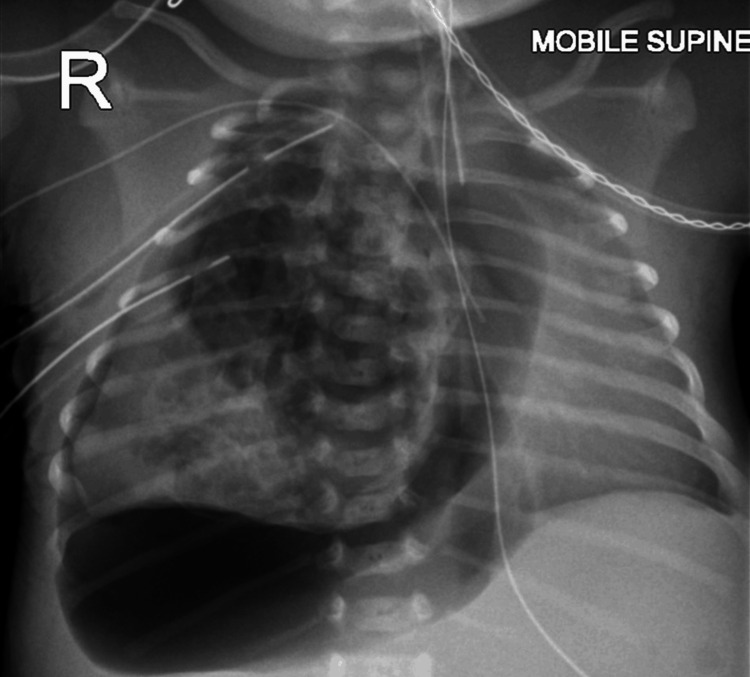
Chest X-ray of the newborn on day 1 of life showing a right-sided pneumothorax A large right tension pneumothorax with cystic lucencies in the collapsed right lung, consistent with the known CPAM. The left lung is partially aerated. CPAM, congenital pulmonary airway malformation

The female patient was delivered at Chelsea and Westminster Hospital via elective cesarean section at around 39 weeks, weighing 2,480 grams. Due to cardiovascular and respiratory instabilities during resuscitation, she was immediately intubated, mechanically ventilated with inhaled nitric oxide at 20 ppm, and transferred to the neonatal intensive care unit. A chest X-ray revealed a right-sided pneumothorax (Figure 1), prompting the insertion of two 10 Fr chest drains (in the fourth intercostal space and apically), which were connected separately to underwater seals at -0.8 kPa suction.

**Figure 2:**
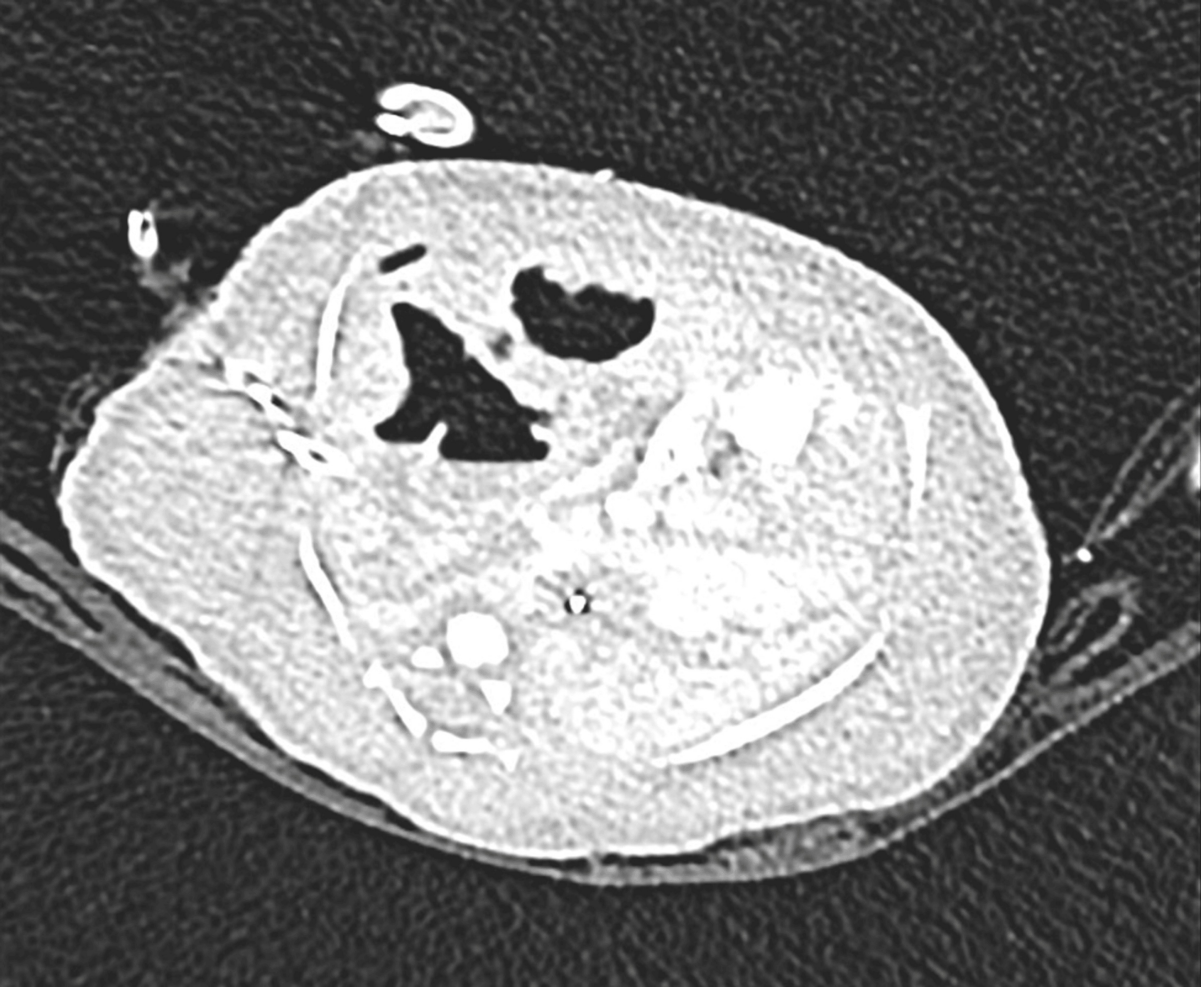
Chest CT of the newborn showing a right middle lobe CPAM A large right middle lobe congenital cystic malformation occupies much of the right hemithorax, with a significant contralateral mediastinal shift. A relatively small patent ductus arteriosus is also noted. CPAM, congenital pulmonary airway malformation

On the second day of life, due to respiratory and cardiovascular instabilities while on mechanical ventilation, the patient was placed on veno-arterial extracorporeal membrane oxygenation (VA/ECMO). A chest CT revealed a right middle lobe CPAM with a significant contralateral mediastinal shift, which explained the patient’s instability (Figure 2). A simultaneous head CT showed no structural abnormalities, consistent with the earlier bedside transcranial ultrasound.

Under VA/ECMO support on the second day of life, a complete resection of the right middle lobe was performed via a right posterolateral thoracotomy. Additionally, a right upper lobe anterior segmentectomy was undertaken due to a persistent air leak observed in the operating theater. Prior to closure, two 10 Fr chest drains were inserted and connected to separate Thopaz systems set at -0.8 kPa suction. Postoperatively, piperacillin/tazobactam and teicoplanin were immediately started and later switched to cefotaxime and gentamicin based on microbiological advice due to rising CRP levels. Gentamicin was discontinued after three days, while cefotaxime was stopped after five days.

Postoperatively, the patient was transferred to the pediatric intensive care unit (PICU) and remained on ECMO/VA support for an additional two days before being decannulated. She was kept intubated for four days, after which she was extubated and placed on high-flow nasal cannula oxygen for four days. She was reintubated due to respiratory deterioration caused by the inspiratory paradoxical motion of the right hemidiaphragm and remained intubated for a total of 39 days. Piperacillin/tazobactam was restarted after reintubation for four days, having been previously stopped due to negative cultures. At one month of age, during the reintubation period, an echocardiogram, cardiac CT, and perfusion study were performed, revealing no abnormal findings except for narrowing of the left pulmonary artery during the perfusion study. However, this narrowing did not limit left pulmonary perfusion. At two months of age, the patient was extubated for the second time and placed on high-flow nasal cannula oxygen, eventually being weaned to room air. Chest drains were removed one at a time after showing no air leak for more than 24 hours and less than 5 ml/kg/hr of output over a 24-hour period.

**Figure 3:**
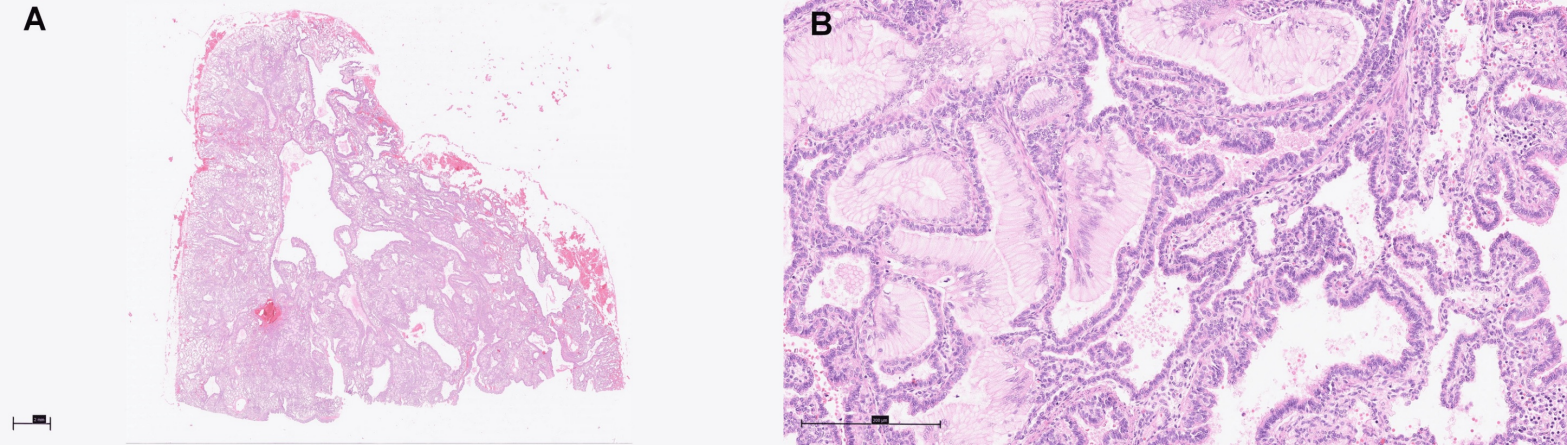
Mucinous cell clusters/mucinous adenocarcinoma arising from type 1 congenital pulmonary adenomatoid malformation (A) Cysts are lined by ciliated respiratory-type epithelium, interspersed with lung parenchyma showing developmental arrest at the late saccular to alveolar stage. (B) The cystic lesion exhibits multifocal involvement by mildly atypical mucinous cells with foveolar-type morphology and a pure lepidic growth pattern.

The pathology report of the resected lesion revealed type 1 CPAM with coexistent mucinous proliferation, interpreted as IMA (pT1b) with free surgical margins (R0). The invasive mucinous component measured at least 15 mm, with no invasion of the visceral pleura. Tumor cellularity was low to intermediate, and tumor content was assessed at 25%. Molecular analysis of the resected lesion identified a KRAS (G12D) mutation. The tumor was negative for EGFR, ALK, BRAF, ERBB2, MET, TP53, CDKN2A, and RB1 genes, consistent with the literature (Figure 3) [13].

**Figure 4:**
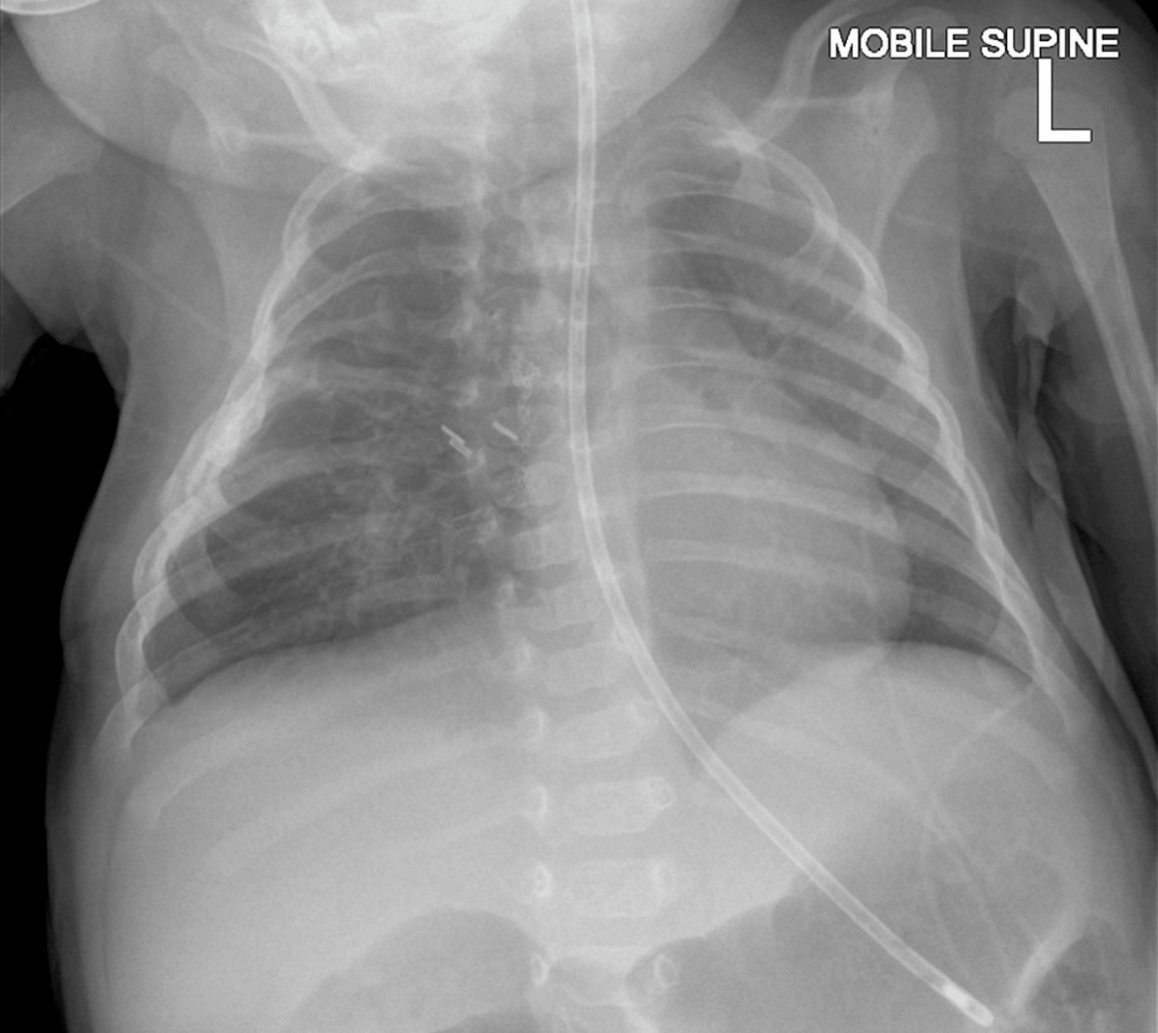
Postoperative chest X-ray The chest deformity at the site of the thoracotomy remains stable in appearance. The lungs are well-aerated, and the cardiomediastinal contours are normal.

The patient was discharged after 71 days in the PICU/HDU and eight days of ward-level care. At discharge, she was able to safely take a daily dose of 10 ml of formula milk with no overt signs of discoordination, and her chest X-ray showed a satisfactory, stable post-surgical appearance (Figure 4). She continued to receive the rest of her nutritional requirements through nasogastric tube feeding. Anti-reflux medications and a weaning regimen from sedatives were prescribed for the post-discharge period. The patient was discharged with planned follow-up by cardiology, respiratory, dietetics, speech and language therapy, as well as pediatric oncology, at various intervals to monitor progress. The gyne-oncologist advised that no follow-up was required for the mother, as there was minimal risk of malignant cell seeding from the pleuro-amniotic shunt, with no histological evidence of invasion of the visceral pleura.

## Discussion

Although CPAMs are considered rare, they are the most common congenital lung disease, typically detected prenatally between 18 and 20 weeks of gestation [1].

Histopathological examination of the resected right middle lobe in our patient revealed type 1 CPAM with IMA. Type 1 CPAM is the most common type, with roughly one-third of cases showing papillary growth of mucinous cells on the cyst surface [7]. There is significant debate regarding the appropriate terminology for these mucinous cell proliferations and whether they should be classified as neoplastic or nonneoplastic lesions. Some argue against labeling these lesions as malignant or premalignant, citing a lack of malignant behavior if complete resection is achieved [14]. However, a study by Chang et al. demonstrated that recurrence can occur several years after initial surgical resection in their series of 37 patients [15]. Furthermore, mucinous proliferations can spread directly through the alveolar parenchyma adjacent to cystic areas, as shown by Summers et al., who reported contralateral lung metastasis of mucinous adenocarcinoma in an eight-year-old patient with a well-differentiated multifocal lesion following lobectomy [16].

Molecular analysis of the mucinous cells in our study revealed KRAS G12D mutations, which are commonly observed in de novo mucinous adenocarcinomas in adults [17]. This supports the classification of these proliferations as having neoplastic potential. Similar mutations were found in most lesions, as reported by Chang et al., who identified KRAS G12D in 50% of cases and KRAS G12V in 40% of cases, with overall mutations present in 90% of their study [15]. Thus, the molecular findings and the recurrence of these lesions years after initial surgery support using the WHO criteria of AIS and invasive adenocarcinoma for their description and classification [18].

In our case, anatomical resection of the lesion was performed, adhering to the recommended optimal treatment for CPAMs. Nonanatomical resection is associated with a higher incidence of residual disease and recurrence after initial surgery [19]. This approach often makes it challenging to definitively identify the boundary between diseased and normal tissue, particularly given the malignant potential of mucinous cells that can spread through the alveolar parenchyma [15]. A study of 44 CPAM cases with mucinous proliferations found that anatomical resection, such as lobectomy, resulted in better outcomes compared to nonanatomical resection [14].

Our patient experienced severe cardiovascular and respiratory instability from birth, necessitating mechanical ventilation and ECMO support for over two months, with only brief periods of clinical improvement. Clinical outcomes for CPAMs depend on several factors, including prenatal diagnosis of nonimmune hydrops, symptomatic presentation at birth, and the size of the lesion [20].

In utero transmission of maternal cancers to infants is an extremely rare event, with an estimated incidence of 1 case per 500,000 mothers with cancer [21]. The proposed routes of transmission are transplacental hematogenous spread or direct spread during vaginal delivery. Direct spread has been documented in cases of fetal lung cancer transmitted from maternal cervical cancers [22]. Although fetal-to-maternal malignant cell seeding has never been documented, there is a theoretical risk of direct seeding of fetal malignant cells into the mother via pleuro-amniotic shunting. However, histopathological examination of the visceral pleura and pleural fluid in our case revealed no malignant cells, further disproving this theoretical risk.

## Conclusions

CPAMs are rare multicystic lung lesions typically diagnosed antenatally. We present a case of a term female neonate with antenatally diagnosed CPAM who required pleuro-amniotic shunting at 22 weeks of gestation and successfully underwent definitive lung resection with ECMO support. To our knowledge, this is the first documented case of CPAM type 1 with IMA associated with pleuro-amniotic shunting in the second trimester. Further research is needed to evaluate the risk of malignant cell seeding through the pleuro-amniotic shunt and the implications for maternal follow-up.

## Appendices

 Appendix A

**Table 1:** Stocker classification describing the five major types of CPAM Source: Mehta and Sharma (2024) [23] CPAM, congenital pulmonary airway malformation

Stocker classification	Origin	Appearance	Prognosis
Type 0	Trachea or bronchi	Bronchial-type airways with cartilage, smooth muscles, and glands separated by mesenchyme	Incompatible with life
Type 1	Bronchi	Cysts are usually multiloculated, within one lobe and lined with pseudo-stratified columnar epithelium	Excellent prognosis following resection. Rarely undergoes malignant transformation
Type 2	Bronchiolar regions	Multiple small cysts with no mass effect	Good prognosis, no malignant potential
Type 3	Bronchiolar regions	Typically solid versus cystic, usually devoid of pulmonary arteries within the lesion	Good prognosis, no malignant potential
Type 4	Acinar structures of the lung	Multiloculated, peripheral, thin-walled cysts lined with type 1 or type 2 alveolar cells	Strong association with pleuropulmonary blastomas
